# Artificial Intelligence in Upper Gastrointestinal Endoscopy: Current Evidence, Practice, and Future Directions

**DOI:** 10.7759/cureus.96657

**Published:** 2025-11-12

**Authors:** Ahmed Alemam, Rezuana Tamanna, Mohamed Ali, Nazeer Ibraheem, Ahmed Swealem

**Affiliations:** 1 General Surgery, Leicester Royal Infirmary, University Hospitals of Leicester, Leicester, GBR; 2 General Surgery, Craigavon Area Hospital, Craigavon, IRL; 3 Surgery, Hywel Dda University Health Board, Carmarthen, GBR; 4 Urology, New Cross Hospital, The Royal Wolverhampton NHS Trust, Wolverhampton, GBR; 5 Orthopedics, Southmead Hospital, North Bristol NHS Foundation Trust, Bristol, GBR

**Keywords:** ai, artificial intelligence, cade, esophageal neoplasia, gastric cancer, helicobacter pylori, upper git endoscopy

## Abstract

Artificial intelligence (AI) is advancing upper gastrointestinal endoscopy (UGIE), ranging from manual, variable workflows to assisted, quality-controlled examinations. Computer-aided detection (CADe) and diagnosis (CADx) systems can help in identifying anatomy, reducing blind spots, and shifting random biopsies toward targeted sampling. Across Barrett’s neoplasia, esophageal squamous cell carcinoma, early gastric cancer, and *Helicobacter pylori* assessment, deep learning models may match or approach expert performance while accelerating image review. Architectures spanning convolutional neural networks to reinforcement learning tools demonstrate high sensitivity and specificity for lesion detection, invasion-depth estimation, and characterization. However, routine adoption requires rigorous, prospective, multicenter validation; mitigation of dataset bias and domain shift; attention to false positives, alarm fatigue, and workflow design; and training that prevents over-reliance on AI. With human-in-the-loop oversight, interpretable outputs, and cost-conscious deployment, AI can standardize inspections, improve diagnostic confidence, enhance training, and deliver better patient outcomes overall without displacing clinical judgment. In this narrative review, we aim to summarize these recent advancements, discuss the performance of AI across key upper gastrointestinal applications, and critically evaluate the practical challenges and future directions for its clinical implementation.

## Introduction and background

In many medical specialties and fields, artificial intelligence (AI) is expanding quickly, raising concerns about both its potential and limitations. Since AI is now utilized in fields such as radiology, cancer, and the development of algorithms to direct patient care, these concerns are particularly pertinent to the medical field [1-3].

Although esophagogastroduodenoscopy (EGD) is the accepted and widely adopted technique for identifying upper gastrointestinal (UGI) disorders, there is still a significant chance of misdiagnosis, mostly because of human cognitive and technological problems of limitations [4]. To avoid missing lesions, modern healthcare depends on thorough quality evaluation of endoscopic procedures. Endoscopists must carefully examine all stomach areas and landmarks in order to accomplish this [5].

Finding any problem or lesion that is present is the aim of upper gastrointestinal endoscopy (UGIE). However, because early lesions can be subtle and challenging for the human eye to detect, complete mucosal exposure alone may not guarantee the detection of every lesion. In these circumstances, AI could improve lesion detection [6,7].

AI is mostly used in endoscopy for computer-aided detection (CADe) and computer-aided diagnosis (CADx) [8]. AI systems ensure comprehensive examinations by identifying significant anatomical landmarks in the UGI disorders [9,10]. CADx systems can help endoscopists with less experience by standardizing observations and reducing differences between observers [11]. In this review, we aimed to summarize the current literature reporting the use of AI-assisted endoscopy.

## Review

Methodology

We conducted a narrative review to synthesize current evidence on AI in UGIE. Between January 2006 and June 2024, we searched MEDLINE/PubMed, Embase, Scopus, Cochrane Library, and IEEE Xplore using combinations of the following terms: “upper GI endoscopy”, “esophagogastroduodenoscopy”, “artificial intelligence”, “deep learning”, “CADe”, “CADx”, “esophageal squamous cell carcinoma”, “gastric cancer”, and “*Helicobacter pylori*”. We limited inclusion to English-language human studies evaluating AI for UGIE quality assurance, lesion detection/characterization, staging, or *H. pylori* assessment, including randomized trials, prospective/retrospective cohorts, cross-sectional validations, and meta-analyses.

We excluded colonoscopy-only studies, pediatric-only cohort studies, non-endoscopic imaging studies, purely technical papers without clinical performance, case reports, and conference abstracts without full data. We screened titles/abstracts, reviewed full texts, and extracted study aims, dataset composition, model families, validation design, performance metrics (e.g., sensitivity, specificity, AUC), and human-AI comparisons. Disagreements were resolved by discussion. Risk-of-bias was qualitatively assessed (dataset bias, domain shift, leakage, and generalizability), recognizing that a narrative synthesis precludes formal meta-analysis. Where available, we prioritized multicenter, prospective evidence. Findings were organized by UGIE task: anatomical landmarking/quality, lesion detection and CADx, depth staging, and *H. pylori *detection, with attention to interpretability, workflow, and clinical implications.

We did not perform a formal, tool-based risk-of-bias assessment (e.g., QUADAS-2/QUADAS-AI, PROBAST/PROBAST-AI). This decision was driven by substantial heterogeneity in study designs, targets (quality metrics, detection, CADx, staging, *H. pylori*), outcome definitions, and reporting standards across the UGIE AI literature, which precluded consistent item-level scoring and meta-analytic pooling. Our objective was a narrative synthesis and landscape mapping rather than effect-size estimation. Instead, we conducted a structured qualitative appraisal focusing on dataset representativeness (class balance, lesion prevalence, multi-center diversity), ground-truthing and blinding, partitioning and leakage safeguards, external validation, reader-study design, and clinical applicability. We acknowledge this as a limitation and note that future updates will incorporate standardized AI-appropriate tools as consensus and reporting mature.

Technical benefits of AI in upper GI endoscopy

Detecting Anatomical Landmarks

AI has shown promise in identifying and classifying locations in the upper gastrointestinal (GI) tract. Takiyama et al., for instance, developed a convolutional neural network (CNN) especially to recognize anatomical locations in EGD images [12]. Wu et al. developed the Wisense AI system, which utilizes reinforcement learning to classify 26 EGD sites. This system also monitors blind spots in real time, achieving a 90.02% accuracy rate and demonstrating substantial advancements in real-time applications [13,14]. Others developed an AI-powered quality control system for EGD using CNNs, trained on 2,599 images from 250 surgeries. It achieved 97.58% accuracy and 97.42% sensitivity in classifying EGD images into eight locations [15].

Enhancing Visualization to Limit Blind Spots

WISENSE, a real-time quality improvement system, was developed by Wu et al. to reduce blind spots during EGD surgery. The system, trained on 34,513 stomach images, demonstrated 90.40% accuracy in detecting blind spots in live EGD videos. A single-center randomized controlled trial (RCT) showed a significant decrease in missed spot rates, with the WISENSE group having 5.86% compared to 22.46% in the control group. Additionally, WISENSE improves the quality of daily endoscopy by automatically generating photo files [13].

A prospective, single-blind RCT involving 437 participants investigated the impact of AI assistance across endoscopy procedures: unsedated conventional EGD (c-EGD) and sedated c-EGD. Each procedure group was further divided based on the use of an AI aid. Notably, AI-assisted groups demonstrated a significantly lower blind-spot rate (3.42%) compared to control groups (22.46%) across all modalities. The most substantial improvement was observed within the sedated c-EGD cohort [14].

Enhancing the Detection Rate of Endoscopic and Navigation-Assisted Biopsy

AI systems can assist endoscopists in transitioning from random to targeted biopsies, thereby enhancing the detection rate of endoscopic lesions. De Groof et al. developed a deep learning (DL) CADx system for early esophageal tumor detection, achieving 89% accuracy, 90% sensitivity, and 88% specificity in classifying neoplasms or non-dysplastic Barrett's esophagus. It also accurately identified optimal biopsy locations in 97% and 92% of cases in two validation datasets [16].

**Figure 1:**
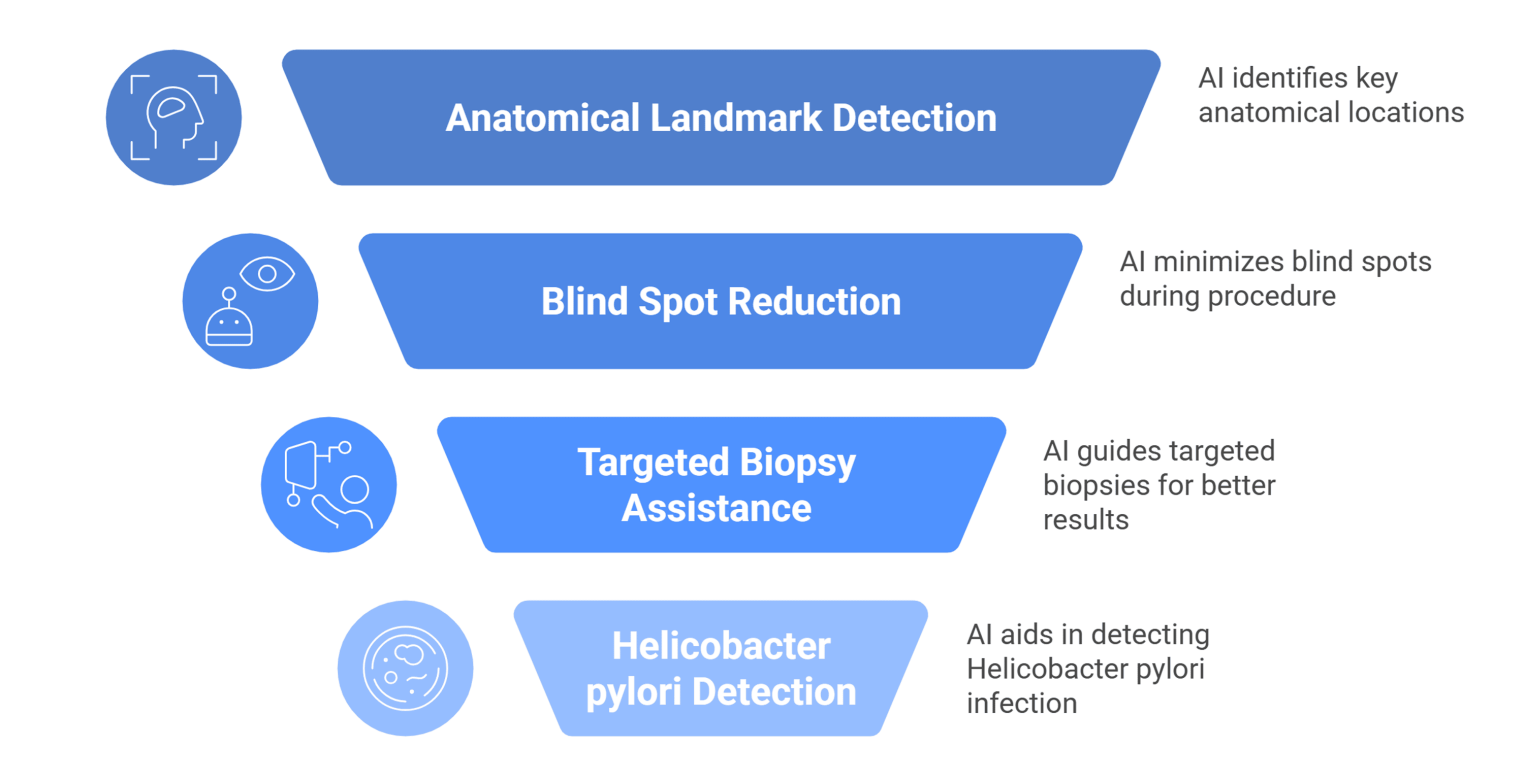
Technical benefits of AI in upper GI endoscopy Figure created by article authors summarizing the benefits of AI-assisted upper GI endoscopy Source: [12-17].

Shichijo et al. developed a CNN for detecting *H. pylori* by classifying the stomach's anatomical regions. Using 32,208 endoscopic images, the authors trained 22-layer GoogLeNet CNNs to diagnose *H. pylori* infection and tested them on 11,481 images from 397 patients. A basic model achieved 81.9% sensitivity, 83.4% specificity, and 83.1% accuracy. A location-aware model, trained on eight gastric regions, improved performance (AUC 0.93): sensitivity 88.9%, specificity 87.4%, accuracy 87.7%, classifying the test set in 3.2 minutes. In comparison, 23 endoscopists averaged 79.0% sensitivity, 83.2% specificity, 82.4% accuracy, and ~230 minutes; board-certified experts reached 88.6% accuracy. Overall, the location-aware CNN was significantly more accurate than endoscopists by 5.3% (95% CI 0.3-10.2), suggesting clinical utility (Figure 1) [17].

Identifying and describing lesions in the upper GI tract

The automatic detection of lesions within the gastric tract has attracted significant research interest, driving numerous efforts to adapt and apply advanced DL models for image classification and object detection to the analysis of UGIE videos [10]. Recent meta-analyses indicate that these approaches consistently achieve high accuracy [5,9]. Another meta-analysis included relevant databases up to July 2020 and investigated the diagnostic performance of AI in identifying and characterizing upper GI tract lesions. An AI system has been shown to accurately detect UGI tumor lesions, including esophageal squamous cell neoplasia (ESCN), Barrett's esophagus-related neoplasia (BERN), and gastric adenocarcinoma (GCA), with a 90% sensitivity [18].

A study tested how well endoscopists detect upper GI tumors and benchmarked them using an AI validation process and framework. The AI achieved AUCs of 0.86 (BERN) and 0.90 (ESCN), suggesting that such validation could become a standard for evaluating clinician performance, given that endoscopists’ tumor-detection accuracy in the study was only moderate [9].

Because intestinal-type gastric cancers are so common, it is essential to find stomach lesions during UGIE. These cancers frequently arise from *H. pylori*-induced chronic inflammation, which results in intestinal metaplasia (IM) and gastric atrophy (GA). Given their increased risk of cancer, routine endoscopy is essential for the early and accurate detection of these precancerous lesions [19-22]. Within this context, recent work has focused on algorithms that can flag suspicious frames in real-time and help endoscopists triage which areas require closer inspection or biopsy.

**Figure 2:**
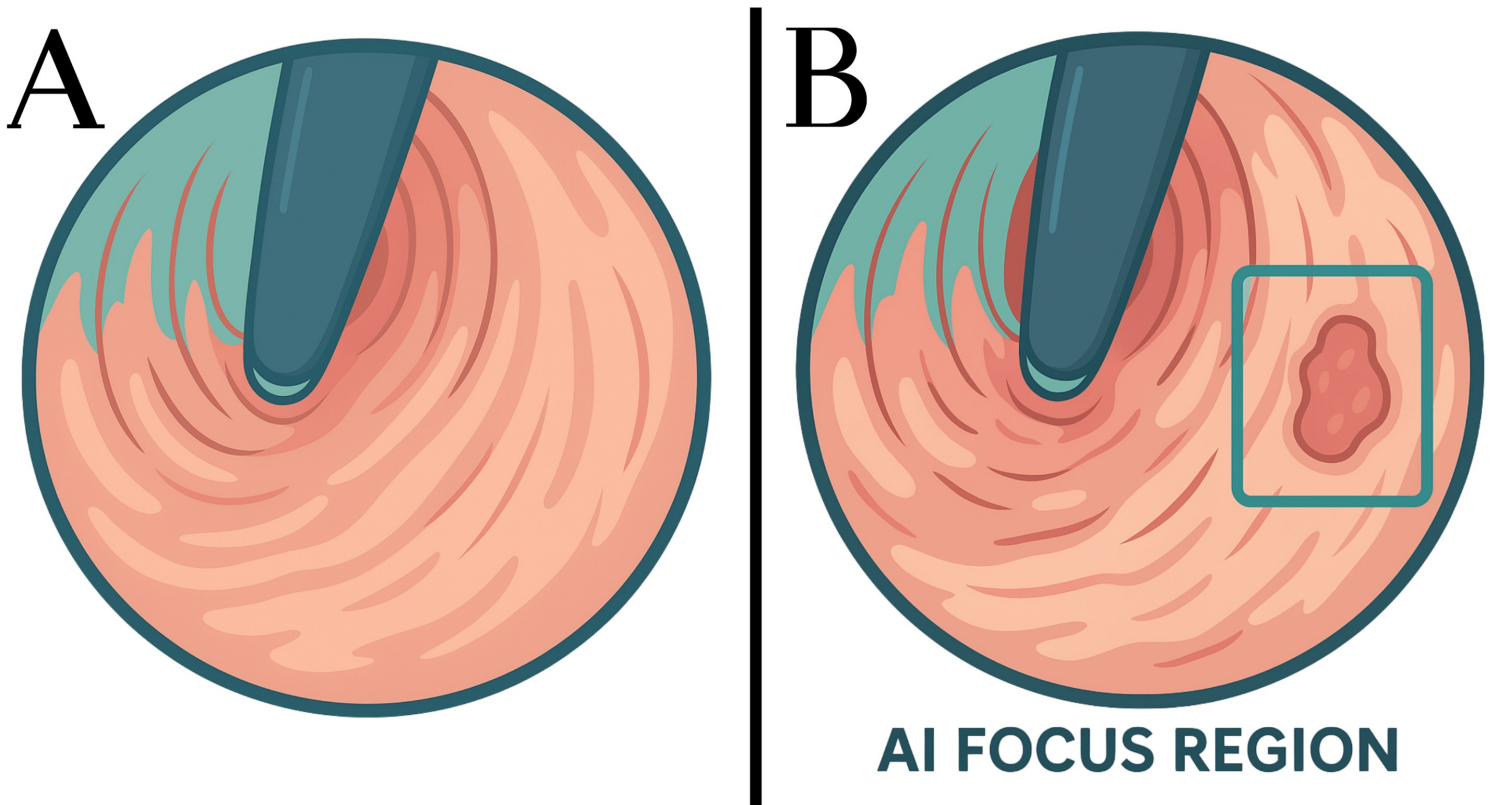
How AI-assisted endoscopy helps endoscopists A cartoon figure showing how AI can identify and highlight lesions. (A) An early gastric cancer lesion missed by an endoscopist and (B) correctly identified by the convolutional neural network in the bounding box regression mode. Figure designed with Canva and Biorender, based on sources [24-26]

Methodologically, two complementary problem framings dominate (i) event (frame-level) detection, which classifies each UGIE image as “lesion present/absent,” and (ii) object detection, which adds spatial localization via bounding boxes [23,24]. Event detection typically relies on established CNN backbones - DenseNet, VGG-16, and ResNet-50 - while comparative experiments have also explored Xception, NASNet, and EfficientNet-B4, with the latter yielding the best performance for IM detection in one study [7,24-27]. Notably, one VGG-16 approach compared standard cross-entropy training with a novel loss that incorporated Grad-CAM terms to nudge the network toward fine-grained, lesion-relevant features when detecting early gastric cancer (EGC) (Figure 2) [26].

Applications of AI in the Detection of Helicobacter pylori Infection 

*Helicobacter pylori* is strongly associated with gastric cancer [21]. Eradicating it can lower the risk of gastric cancer [22]. AI technologies offer new ways to detect *H. pylori* infections by identifying differences between infected and uninfected stomach linings. Research shows AI can accurately detect *H. pylori*, with success rates between 74.8% and 91.9% [20,23-26].

AI has been employed in numerous studies to identify infected, non-infected, and post-eradication treatment mucosa, leveraging the extensive and diverse image datasets available for training. Beyond diagnostic accuracy, the speed of AI-based diagnosis has also been investigated. In one study, an AI model analyzed 11,481 images in 194 seconds, achieving 87.7% diagnostic accuracy. In contrast, physicians in the control group took an average of 230.1 minutes for the same task, with an overall accuracy of 82.4% [17].

AI aids endoscopists in rapid image assessment, thereby improving diagnostic accuracy and reducing diagnosis time. This is especially crucial in preventing misdiagnoses caused by physician fatigue [28].

AI-assisted endoscopy in esophageal and gastric cancer

Because advanced esophageal and gastric cancers frequently have a poor prognosis, UGI endoscopic cancer screening is essential. According to reports, the missed diagnosis rate for UGI cancers in the European community ranges from 5% to 11% [18].

Barrett's Neoplasia and Depth of Cancer Invasion

Early research on CADx shows diagnostic accuracy comparable to that of histopathology. For example, CADx obtained 82% sensitivity, 74% specificity, and 83% accuracy in a pre-labeled database of endoscopic optical coherence tomography [29]. Further investigation and analysis have demonstrated a consistent and similar level of performance in a relatively small validation sample, comprising a total of 44 participants [30]. This outcome suggests the robustness of the findings, even within a more constrained dataset, and supports the initial conclusions drawn from the primary study. While the sample size is modest, the consistent performance indicates a strong potential for the observed effects to generalize to a broader population, pending further large-scale validation studies.

A Japan-developed CADx system for esophageal squamous cell carcinoma (ESCC) depth assessment made by Nakagawa et al accurately separated superficial (mucosal or microinvasive submucosal) from deeply invasive submucosal disease, achieving 90.1% sensitivity, 95.8% specificity, and 91.0% accuracy, performing on par with 16 expert endoscopists [31].

Other CADx systems demonstrated superior performance in detecting ESCCs, identifying 95.5% (279/291) of cases in test images within 10 seconds. Furthermore, it accurately estimated the depth of infiltration with an 84.1% sensitivity and 80.9% accuracy, surpassing the accuracy of 12 out of 13 endoscopic experts [32]. Zhu et al. developed a CNN algorithm to evaluate the depth of gastric cancer invasion. This system, trained on 790 endoscopic images and verified with 203, demonstrated an accuracy of 89.2%, a sensitivity of 74.5%, and a specificity of 95.6% [33]. Kanesaka et al. created a CADe tool that utilizes magnified narrow band imaging (NBI) images to identify and delineate cancerous and non-cancerous gastric lesions. This tool demonstrated high accuracy (96.3%), sensitivity (96.7%), and specificity (95%) [34].

Miyaki et al. developed a support vector machine (SVM)-based system to identify gastric cancer using BLI endoscopy. Trained with 587 cancer and 503 normal tissue images and then validated with 100 early gastric cancer images, the system showed significantly higher SVM output values for cancerous lesions (0.846 ± 0.220) compared to red lesions (0.381 ± 0.349) and surrounding tissue (0.219 ± 0.277). Undifferentiated cancer also had a higher mean SVM output than differentiated cancer [35].

Squamous Cell Neoplasia

In lower-income nations, ESCC constitutes as much as 90% of esophageal cancers. Its prognosis is bleak, with an overall five-year survival rate of 18%, plummeting to less than 5% if distant metastases are present at the time of diagnosis [36]. AI systems demonstrate superior sensitivity in detecting lesions compared to experienced physicians (100% vs. 92%) while maintaining comparable specificity for noncancerous lesions (63% vs. 69%). Furthermore, DL-based CADx tools have shown high accuracy in recognizing early ESCC under white-light endoscopy (WLE). These tools surpass non-expert endoscopists in accuracy and achieve similar accuracy to expert endoscopists (97.6% vs. 88.8% and 77.2%, respectively) [37].

A high-resolution microendoscopy system with a tablet interface was used for automated diagnosis of esophageal squamous mucosa. This system facilitated morphometric nuclear analysis, achieving an overall sensitivity of 93%, specificity of 82%, and an AUC of 0.87 [38].

CNNs have been tested on manually generated regions of interest for detecting NBI intra-papillary capillary loops. A more extensive study, utilizing 8,428 WLE images, showed a sensitivity of 98% and a negative predictive value (NPV) of 95%. This study also highlighted the rapid analysis capability, processing 1,118 images from the test database in 27 seconds [39]. A CNN trained with GoogLeNet for endocytoscopy showed a 92.6% sensitivity when tested on a database of 55 patients, 27 of whom had neoplasia [40].

A retrospectively trained CNN demonstrated excellent performance in detecting early SCC and differentiating it from inflammation. Tested on 948 images, the CNN achieved a sensitivity of 97% and a specificity of 94% [41]. When tested on unseen videos, CNNs initially trained on static images successfully detected SCC in 80% of patients (8 out of 10). However, the positive predictive value was only 42.1%, indicating a high false positive rate that could potentially be reduced with additional training [42].

Gastric Cancer

Gastric cancer ranks among the top five most prevalent cancer diagnoses worldwide and is the third leading cause of cancer-related deaths. Detecting premalignant lesions early is crucial for mitigating the disease's impact. Endoscopic examination remains the gold standard for evaluating the gastric mucosa. A recent systematic review and meta-analysis highlighted the growing significance and positive influence of AI in gastric cancer, indicating its increasing role in the present and future [43].

Hirasawa et al. pioneered the development of the first AI system for gastric cancer detection utilizing DL. This system demonstrated high sensitivity, achieving 92.2% for lesions measuring 5 mm or smaller and 98.6% for lesions measuring 6 mm or larger. However, its positive predictive value (PPV) was only 30.6% due to the misdiagnosis of mild-to-moderate chronic atrophic gastritis (CAG) and IM lesions [24]. Another study found that a DL approach significantly outperformed human experts in assessing CAG [44].

Horiuchi et al. also developed an AI system that achieved 85.3% accuracy, 95.4% sensitivity, and 71.0% specificity in differentiating gastric cancer from gastritis [45]. Wu et al. evaluated a DL system for gastric cancer detection, reporting a sensitivity of 94.0%, specificity of 91.0%, accuracy of 92.5%, positive predictive value (PPV) of 91.3%, and NPV of 93.8% [46]

Li et al. developed a CNN-based AI model to differentiate between noncancerous and early gastric mucosal lesions during magnifying endoscopy (ME). This system demonstrated superior diagnostic capabilities for early gastric cancer compared to non-expert endoscopists, achieving a sensitivity of 91.1%, specificity of 90.6%, and accuracy of 90.9% [47].

Using the CNN DenseNet, Zhang et al. achieved a diagnostic accuracy, sensitivity, and specificity of 0.94, 0.95, and 0.94, respectively, for identifying CAG lesions. The classification accuracy for mild, moderate, and severe CAG was 0.93, 0.95, and 0.99, indicating higher detection rates for moderate and severe cases [48].

Multiple multicenter studies have found that AI combined with ME with NBI (ME-NBI) has a diagnostic performance comparable to that of senior endoscopists and superior to that of junior endoscopists. Notably, endoscopists' diagnostic accuracy significantly improved after consulting the AI system's results, highlighting a practical application of AI in daily practice [49].

Limitations and future directions

Incorporation of CADe into routine clinical practice hinges on several considerations, including the potential for bias during algorithm training or validation as unbalanced datasets, site-specific imaging artifacts, or inadvertent data leakage; the nature of its interaction with human endoscopists in real time (how overlays, prompts, and alerts shape attention, workflow, and decision-making); and the clinical implications of false-positive results such as unnecessary biopsies, prolonged procedure time, added costs, and heightened patient anxiety [50].

Achieving reliable AI results necessitates optimal inspection techniques, potentially involving extended inspection times and specialized operator expertise, such as the use of magnification. These conditions are not consistently met in clinical practice. While numerous studies have explored AI's role in detecting and diagnosing upper GI cancer, most are uncontrolled and do not operate in real-time during endoscopy [51].

Future endoscopists training with AI may become overly reliant on it, potentially hindering their cognitive diagnostic and therapeutic abilities. AI should enhance, not replace, human cognition. While AI shows promise in endoscopy, its DL "black boxes" can lead to unpredictable, unexplainable errors. Therefore, AI is best used for preliminary screening, identifying areas of interest, and predicting histology, with humans making final decisions. Although AI may eventually surpass expert diagnostic skills, the endoscopist remains responsible for missed cancers or misdiagnoses. AI should augment, not compensate for, poor endoscopic technique [51].

## Conclusions

AI is maturing from promising prototypes to tools that consistently enhance UGIE quality and diagnostic confidence. Across tasks, such as landmarking, blind-spot reduction, lesion detection/characterization, staging, and H. pylori assessment, CADe/CADx match or surpass non-expert performance and often approach experts, while speeding review. Properly integrated, AI augments endoscopists by elevating the completeness of inspection and standardizing assessments, without displacing clinical judgment or accountability.

Translating these gains into routine care requires rigorous, prospective, multi-center evaluations, attention to dataset bias and domain shift, and UI designs that support real-time decisions without alarm fatigue. Human-in-the-loop workflows, transparent interpretability, and cost-conscious deployment are essential, alongside education to avoid over-reliance. With these safeguards, AI can measurably improve outcomes and training while preserving the primacy of skilled endoscopy.
